# Models of fibrolamellar carcinomas, tools for evaluation of a new era of treatments

**DOI:** 10.3389/fimmu.2024.1459942

**Published:** 2024-11-08

**Authors:** Jinjia Song, Mengqi Lu, Zhiying He, Wencheng Zhang

**Affiliations:** ^1^ Institute for Regenerative Medicine, Medical Innovation Center and State Key Laboratory of Cardiology, Shanghai East Hospital, School of Life Sciences and Technology, Tongji University, Shanghai, China; ^2^ Shanghai Engineering Research Center of Stem Cells Translational Medicine, Shanghai, China; ^3^ Shanghai Institute of Stem Cell Research and Clinical Translation, Shanghai, China; ^4^ Postgraduate Training Base of Shanghai East Hospital, Jinzhou Medical University, Jinzhou, Liaoning, China

**Keywords:** fibrolamellar carcinoma (FLC), biliary tree stem cells (BTSCs), tumor-derived models, organoids, DNAJB1-PRKACA fusion gene, heparan sulfate (HS)-oligosacchrides

## Abstract

Fibrolamellar carcinoma (FLC) is a rare but fatal cancer that occurs primarily in young people. There are currently no known effective treatments, although several promising treatments appear to be in development. Genetic studies have confirmed that almost all FLC tumors have a fusion protein marker (DNAJB1-PRKACA) encoded by a fusion gene (DNAJB1-PRKACA); It is currently accepted as a diagnostic criterion for FLCs. Several research teams have established patient-derived xenograft (PDX) FLC models using immunocompromised animals as hosts and patient tissue samples (tumors or ascites) as primary sources for PDX-derived organoids. These FLC organoids are composed of FLC epithelia, endothelial progenitor cells, and stellate cells. CRISPR/Cas9 was used as a gene editing technique to modify mature hepatocytes to obtain *ex vivo* FLC-like cells expressing the fusion gene and/or other mutated genes associated with FLCs. Although these models simulate some but not all FLC features. Drug screening using these models has not proven effective in identifying clinically useful treatments. Genetic studies comparing FLCs to normal maturing endodermal cell lineages have shown that FLCs share genetic signatures not with hepatocytes, but with subpopulations of biliary tree stem cells (BTSCs), hepato/pancreatic stem/progenitor cells that consistently reside in peribiliary glands (PBGs) located in the biliary tree and are sources of stem cells for the formation and postnatal regeneration of the liver and pancreas. Therefore, it is expected that models of BTSCs, instead of hepatocytes may prove more useful. In this review, we summarize the status of the various FLC models and their features, applications, and limitations. They provide opportunities to understand the cause and characteristics of this deadly disease and are models from which effective treatments can be identified.

## 1 Introduction

Fibrolamellar carcinoma (FLC) is named for its unique histological features, particularly the large amount of early lineage stage mesenchymal cells, which are precursors to endothelia and stellate cells, associated with FLC tumor cells (1–4). In contrast to patients with conventional hepatocellular carcinoma (HCC), patients with FLC typically have no clinical history of liver cirrhosis; few have hepatitis virus infections; and they are routinely negative for alpha-fetoprotein (AFP), an indicator of other liver tumors such as hepatoblastoma (5, 6). Currently, surgical resection is the primary clinical treatment for FLCs. The 5-year overall survival rate of FLC patients who underwent the surgical procedure ranged from 30% to 48% (7, 8). However, such treatment is not ideal because surgical resection is not suitable for patients with metastatic disease and FLC tumors are prone to recurrence and metastasis after surgical resection (9–11). Sorafenib, oxaliplatin, 5-fluorouracil, and interferon, which can be used as adjuncts to conventional chemotherapy or targeted therapies in HCC, have also been used in the treatment of patients with FLC, although with limited, if any, success; they have failed to improve long-term survival (12). It is suggested that precise immunotherapy or immunotherapy combined with chemotherapy that directly targets FLC tumor-associated proteins offers more logical strategies for effective treatment of FLCs and is increasingly the focus of researcher. However, associated clinical trials of immune checkpoint inhibitors had no impact on disease progression, and clinical trials of vaccination failed in most patients and showed only an isolated response. Therefore, new treatment methods are still in development, which requires further research.

## 2 Genetic signatures and mutations associated with FLC

**Figure 1:**
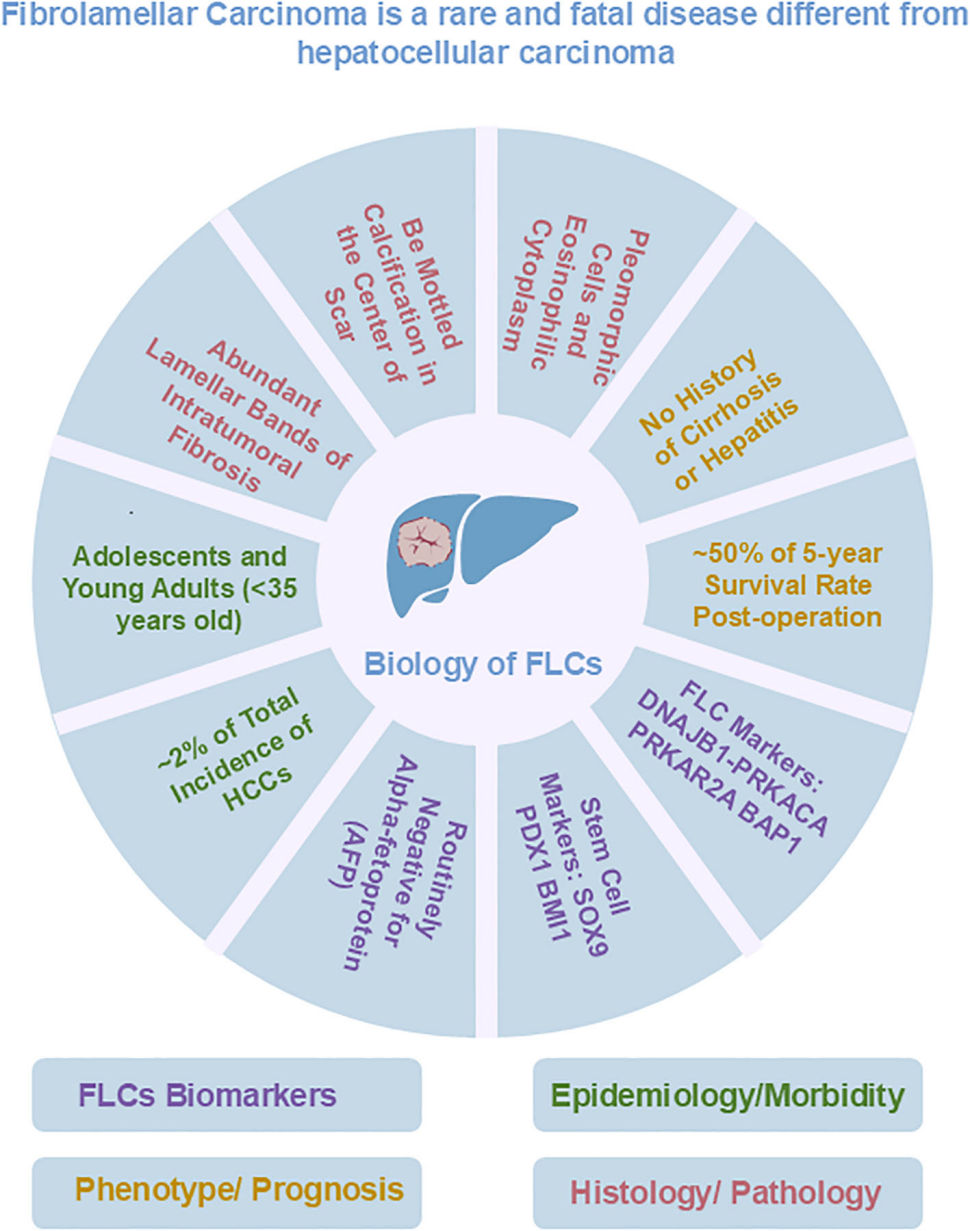
Overview of known biological features of FLC.

**Figure 2:**
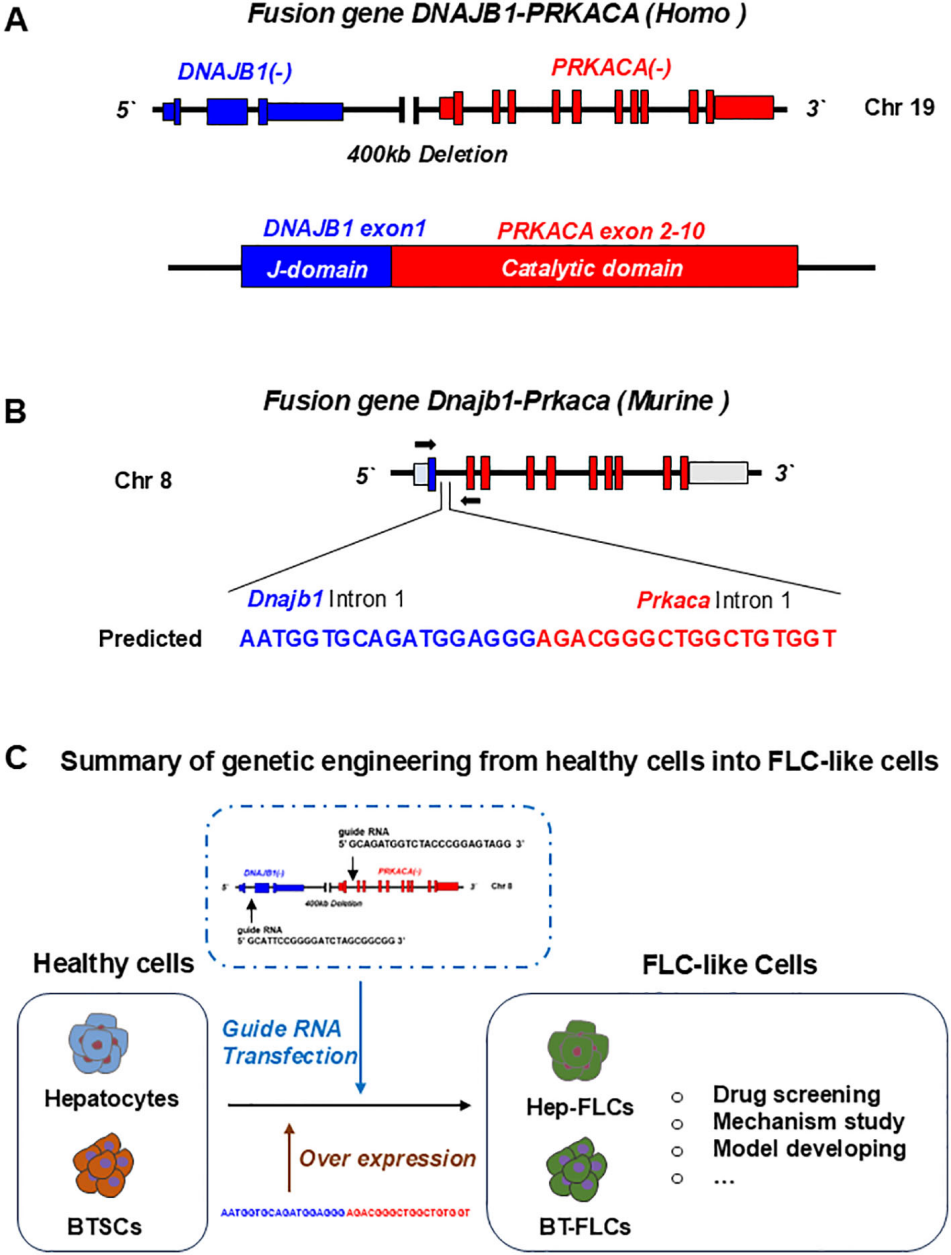
Gene editing of fusion genes in normal liver or cells for developing FLC models. **(A)** In human, fusion gene occurs on chromosome 19, when a 400Kb deletion occurs, leading to the fusion of DNAJB1 and PRKACA. The formed DNAJB1::PRKACA fusion gene can encode the DNAJB1-PRKACA fusion protein to activate protein kinase A by dysregulation of the catalytic portion of the protein. **(B)** The fusion gene can be induced by CRSPR-Cas9 on chromosome 8 in normal mice. **(C)** Normal cells including hepatocytes, biliary tree stem cells (BTSCs) or fetal liver cells can be genetic engineered into FLC-like cells by endogenic expression DNAJB1::PRKACA fusion genes or by over expressing of fusion cDNAs.

The DNAJB1-PRKACA fusion gene is one of the markers of fibrolamellar carcinoma. The fusion of this gene is caused by a heterozygous deletion of approximately 400 kb on human chromosome 19 (**Figure 1**). The resulting DNAJB1-PRKACA fusion transcript is thought to activate protein kinase A through dysregulation of the catalytic portion of the protein (**Figure 2**). Activation of protein kinase A is also a characteristic feature of FLC. Protein kinase A consists of catalytic and regulatory subunits. Among them, PRKACA encodes the catalytic subunit and PRKAR1A encodes the regulatory subunit of protein kinase A. Honeyman et al. first identified this chimeric RNA, DNAJB1-PRKACA, which is predicted to encode a protein containing the amino-terminal domain of DNAJB1, a homolog of the molecular chaperone DNAJ fused in frame to PRKACA. PRKACA is the catalytic domain of protein kinase A and has been shown to be expressed in FLC but not in the adjacent normal liver, suggesting that this genetic alteration contributes to tumor pathogenesis (13).

This has also been confirmed by many other teams (13–15). Graham et al. developed an RT-PCR assay and an RNA *in situ* hybridization assay for paraffin-embedded tissues to detect the rearrangements of the PRKACA locus and calculated the total chimeric transcript and wild-type transcripts. Their results showed that the DNAJB1-PRKACA fusion gene is present in all FLC, while it is not detected in other tumor types. Therefore, they concluded that DNAJB1-PRKACA is an extremely sensitive and specific molecular marker for the diagnosis of FLC (16). Meanwhile, the biological function of the DNAJB1-PRKACA fusion gene and whether it is just a typical marker or a potential tumorigenic mechanism have attracted more attention. By using the gene editing tools, Engelholm et al. proved that the DNAJB1-PRKACA gene fusion can induce liver tumorigenesis with histological and cytological features of human FLCs. These features include large polygonal cells with granular, eosinophilic, and mitochondria-rich cytoplasm, prominent nucleoli, and markers of hepatocytes and cholangiocytes (17). Using a similar strategy, Kastenhuber et al. showed that either induction of the endogenous DNAJB1-PRKACA fusion gene by CRISPR/Cas9 or overexpression of the fusion gene cDNAs was sufficient to induce FLC-like tumors in young adult mice. Most importantly, their study revealed that DNAJB1-PRKACA fusion kinase interacts with β-catenin and acts as an oncogenic driving factor during FLC occurrence (18). Graham et al. further found that it is PRKAR1A loss rather than the classical DNAJB1-PRKACA fusion that is the cause of FLCs (**Figure 2**) (19). They identified three individuals with FLCs and a personal history of Carney complex. All three tumors showed the typical morphology of FLC and were positive for arginase, cytokeratin 7 and CD68, while all were negative for PRKAR1A protein expression. Their results suggested that FLC may be part of the Carney complex. In this case, FLCs have inactivating PRKAR1A mutations instead of the DNAJB1-PRKACA fusion gene found in sporadic FLCs, representing alternative possibilities for activating of protein kinase A.

Other teams such as Jessica Zucman-Rossi and her associates at INSERM (Paris, France) identified a homogeneous subgroup of HCC in which the BAP1 gene is inactivated and has similar features to FLCs (20). These tumors are more frequently developed in females without chronic liver disease or cirrhosis. The presence of PKA activation and T cell infiltrates suggest that these tumors could be treated with PKA inhibitors or immunomodulators. In any case, the DNAJB1-PRKACA fusion gene contributes to an increase in protein kinase activity, a key factor in the occurrence of FLC tumors. Consequently, protein kinase inhibitors may have great therapeutic potential for development and application in the treatment of these pancreatic/biliary tumors once a suitable drug is identified and developed.

## 3 The cellular origin of FLC and the use of gene editing techniques to generate FLC phenotypic traits from normal healthy cells

Gene editing technology generally refers to zinc-finger nucleases (ZFNs), transcription activator-like effector nucleases (TALENs), and clustered regularly interspaced short palindromic repeat DNA sequences (CRISPR/Cas9). CRISPR/Cas9 is considered a powerful gene editing tool that can be used to modify genes in various organisms, including humans. The CRISPR/Cas9 system is a natural immune system found in a variety of bacteria, including archaea, to protect against viral invasion (21). By developing CRISPR-associated enzymes (Cas enzyme), they can specifically target and cleave the target sequence to achieve the purpose of gene editing.

Since its first application in gene editing of mammalian cells in 2013 (22, 23), CRISPR tools have been widely developed and applied and have demonstrated their critical value in the field of tumor research. Xue et al. delivered plasmid DNA expressing Cas9 and sgRNA targeting PTEN and TP53 into mouse liver by tail vein injection and directly induced liver tumors. This study proved the feasibility of using CRISPR/Cas9 to directly target liver cancer genes and tumor suppressor genes to construct liver cancer mouse models (24). Subsequently, plasmids carrying Cas9 and multiple sgRNA targeting genes were injected into KRAS mice model using the same method, resulting in the induction of hepatocellular carcinoma and cholangiocarcinoma (25). Currently, CRISPR/Cas9 technology has been applied to the construction of mouse tumor models such as glioblastomas, pancreatic and lung cancers (26–28), providing an important tool for exploring the function of oncogenes and greatly accelerating the process of tumor research.

Previous studies have provided a comprehensive understanding of the molecular characteristics of FLC tumor tissues. However, the impact of FLC mutations on the healthy cells in the liver and the mechanisms by which different genetic backgrounds drive the occurrence of FLC are not yet known (13).

Rüland et al. constructed organoid models of human fetal hepatocytes with different FLC mutation backgrounds, including endogenous DNAJB1-PRKACA^fus^, PRKAR2A^KO^, BAP1^KO^ and BAP1^KO^;PRKAR2A^KO^ organoid lines (15, 29). Transcriptomic comparison of FLC tumors and wild type fetal hepatocytes revealed that the transcriptional profile of the FLC mutant organoids was generally similar to that of FLC tumors with identical genetic backgrounds. This study suggests that FLCs can be derived from normal healthy cells in the liver after the introduction of BAP1 and PRKAR2A mutations (4). Most interestingly, this study found that various FLC mutations led to a certain degree of hepatocyte dedifferentiation, while the co-occurrence of mutations in BAP1 and PRKAR2A can significantly alter the fate of hepatocytes. The hepatocytes with double BAP1 and PRKAR2A mutations underwent de-differentiation to obtain a stem cell-stage, that has a similar phenotype to cholangiocytes or hepatic progenitor-like cells; and can be cultured under condition suitable for cholangiocytes. This indicated that either hepatocytes or cholangiocytes could be the cellular origin of FLC, in any case, they need to be in a de-differentiation stage in order to obtain the FLC feature.

Dinh et al. ran genetic signature study to identify miRNAs which are abnormal in FLC tumors. He applied RNA-seq comparison between FLCs and four cell types representing distinct maturational lineage stages in liver, including human biliary stem cells (hBTSCs), human hepatic stem cells (hHpSCs), human hepatoblasts (hHBs) and human adult mature hepatocytes (hAHEPs) (30). This genetic study brought a different voice to the cellular origin of FLC. The results showed that FLCs have a genetic signature that overlap notably with that of the hBTSCs and to some extent that of hHpSCs or hHBs, and is significantly different from the genetic signature of mature hepatocytes or cholangiocytes (30–32). Indeed, FLCs are unique in being tumors rich in stem cells (more than 70%), while hepatocellular carcinomas are typically composed of a few percent stem cells and cholangiocarcinomas are perhaps 10% to 12% stem cells (4). However, the experimental verification of whether BTSCs expressing fusion genes through gene editing can better simulate the phenotype of FLC and confirm that BTSCs are the cellular source of FLC is still ongoing.

## 4 Organoid models for FLC

Conventional 2D tumor cell lines (monolayer) have proven inadequate to simulate the phenotypic traits of FLCs, which exhibit critical epithelial-mesenchymal cell-cell interactions involving paracrine signaling pathways, in addition, show aberrant mitochondrial and metabolic functions. Organoids, floating aggregates of epithelial stem/progenitor cells and the associated early maturation lineage stages of mesenchymal cells, typically precursors of endothelia and stellate cells, are widely used as more effective models for disease research.

Organoids were routinely used in the early days of cell culture in the 1930s to 1960s, but they faded from use with the advent of methods by which to establish monolayer cell cultures, especially clonal cell lines, and further enhanced in experimental usefulness with plastic cell culture dishes, developments occurring post-World War II with the development of the plastics industries. A return to studies on organoids has occurred during the last ~15 years with the remembrance of the importance of epithelial-mesenchymal cell-cell interactions, of fundamental importance to metazoans, the relevance of cell polarity and three-dimensionality, and their contribution to improvement abilities for analyzing normal and disease states in tissues were called upon (33).

**Figure 3:**
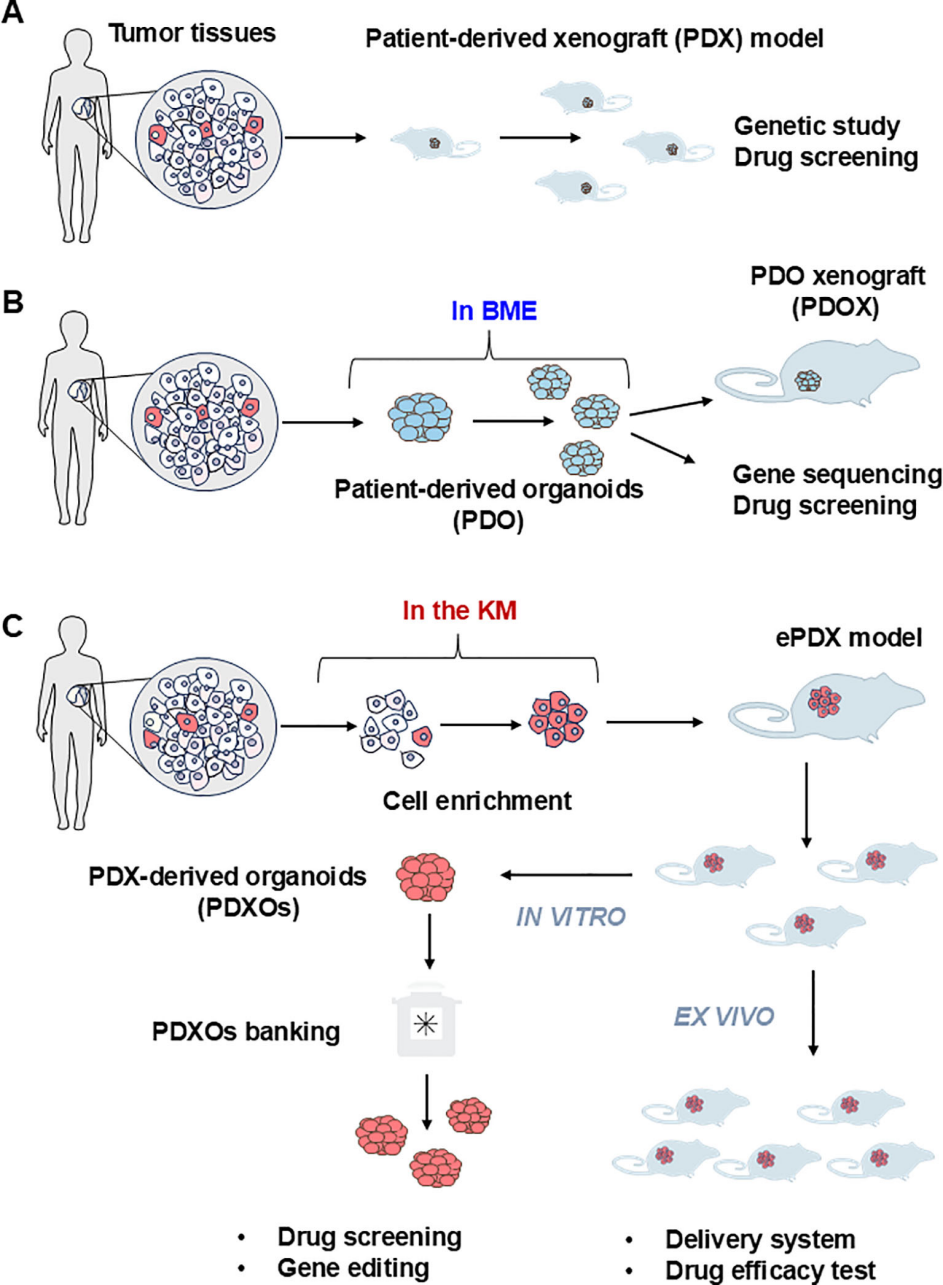
Current procedure of establishing the patient derived models of FLCs. **(A)** The patient-derived xenograft model (PDX) is the first generation of the PDX model for solid tumors. Tumor tissues are mechanically dispersed immediately after dissection with or without enzymatic treatment. The disseminated tumor can be directly injected subcutaneously into the immunocompromised NSG mice to generate the PDX model. Due to the limited amount of tumor imitating cells in the solid tumor tissue, this strategy can only be applied to limited cancer types such as breast cancer. **(B)** Patient-derived organoids (PDO) are currently the most adaptable strategy. Cell mixtures from solid tumor are mixed with basement membrane extract (BME) such as Matrigel with or without purification. The extracellular matrix provides essential nutrients for the growth of epithelial tumor cells to form three-dimensional(3D) spheroids or organoids. PDO can be used immediately or implanted into the NSG mouse to form PDO-derived xenograft model (PDOX) for further *in vivo* assay. **(C)** PDX-derived organoids (PDXOs) were developed by Reid and her associate when they established the first-ever-FLC PDX model (enriched PDX model, ePDX-model) by enriching FLC tumor cells with serum-free Kubota`s Medium (KM), and then injecting the enriched FLC patient tumor cells into NSG mice. This strategy can be applied to any solid tumor associated with cancer stem cells. The PDX-derived organoids can be obtained from the ePDX model any time they are needed for drug screening or genetic studies to develop potential new treatments.

As floating three-dimensional cell aggregates formed *ex vivo* by stem cells of both the epithelia and their mesenchymal cell partners, organoids are more accurate models of both normal and diseased tissues in demonstrating organ-specific and tissue-specific features than any monolayer culture model (34, 35). Tumor organoids prepared directly from human tumor tissue can be used to define their genetic signatures and phenotypic characteristics (**Figure 3**), making them excellent *ex vivo* research tools for normal tissues and organs compared to tumors and for cancer progression (**Table 1**). Referring to the construction methods of organoids from healthy donors, many studies have constructed corresponding organoids from multiple tumors, such as liver (36, 37), prostate (38), lung (39), ovaries (40), and breasts (41), etc. These tumor organoids are widely used for anti-tumor drugs screening, drug toxicity testing, disease modeling, and studying the mutational characteristics of tumors.

**Table 1:** Summary of the currently available FLC *ex vivo* models.

Models	Methods	Advantage	Limitations	Reference	
Patient-derived xenograft hFL-HCC model FLC-TD-2010	Ascites tumor cells derived from FLC patient were cultured briefly (a week or two) in Kubota’s Medium (KM), a serum-free medium designed for endodermal stem/progenitors. Rapid culture selection resulted in organoids of FLC epithelial cells partnered with mesenchymal stem/progenitors (precursors of endothelia and stellate cells). The selected organoids were transplanted into NSG mice	**a. **The FLC organoids expressed the fusion gene DNAJB1::PRKACA and had morphological characteristics of FLCs. **b. **They are tumorigenic in immune-compromised hosts such as NSG mice	The initial tumor formation took a long time (more than 6 months), but thereafter passage took 2–3 months. All transplanted FLC organoids (100%) formed tumors even at very low inoculum concentrations (<100 cells). However, the tumorigenesis rate depended on the number of organoids transplanted; was increased by dietary supplementation (e.g. HGF and VEGF); and by transplantation of organoids embedded in hyaluronan hydrogels.	Tsunekazu Oikawa et al., *Nature Communications* 2015	
Patient-derived xenograft hFL-HCC model	FLC tumor samples were donated from patients (aged 17 to 36 years; 4 women, 2 men) undergoing surgical resection. Tumor tissues were cut into pieces and transplanted subcutaneously, intrahepatically, or under the kidney capsule. A portion of the tumors were digested into single cells and injected into NSG mice intrasplenically, intrahepatically, or subcutaneously.	**a. **The DNAJB1::PRKACA fusion gene and its fusion protein remain stable expression after multiple passages of xenografts. **b. **These PDX models have the typical morphological features of FLCs, such as eosinophilic cytoplasm and areas of fibrosis. **c. **These models express 509 differentially expressed genes of FLCs.	**a. **The success rate for implantation of PDX material into NSG mice was 30-35%. **b. **The culture of PDX materials required several months to a year. **c. **The PDX tumors might not recapitulate the responses of the patients to treatments. **d. **During the evaluation of candidate agents, the response of implanted tumors to the candidate agents may differ from the tumors that has developed spontaneously in a patient.	Gadi Lalazar et al., *Cancer Discovery* 2021
Patient-derived xenograft hFL-HCC model	21 patient-derived organoid lines from 9 patients with FLCs were cultured in either the hepatocyte medium or cholangiocyte medium, including 6 from adjacent normal liver. There are 3 from primary FLCs and 12 from metastatic FLCs.	**a. **They express the fusion gene and the FLC-associated genes and have the morphological characteristics of FLCs. **b. **The organoid lines are from different sites in the patients, including adjacent non-cancerous liver, and from primary and metastatic FLCs.	**a. **The fibrolamellar bands can only be observed in the original tumor tissue. **b. **The transcriptomes of the tumor organoids clustered with each other and with the corresponding tumor tissue, was distinct from that in normal tissue and in normal organoids.	Nicole J.C. Narayan et al., *Stem cell Reports* 2022

Sanford M. Simon and his associates had developed 21 patient-derived organoid lines from 9 patients with FLC, including 6 from adjacent non-malignant liver tissues, 3 from primary FLCs and 12 from metastatic FLCs, with the organoids system developed by Hans Clevers (**Figure 3**, **Table 2**) (42). The metastatic FLC organoid lines were derived from liver, lung, abdominal wall, omentum, ascites, and lymph node metastases at various anatomic locations. The PCR results confirmed that the DNAJB1-PRKACA fusion transcript was specifically expressed in all FLC organoid lines cultured in either hepatocyte medium or cholangiocyte medium. In terms of morphology, these FLC organoids were found to be polygonal and rich in lamellar bands of intratumoral mesenchymal cells. Through transcriptomic analysis, the FLC organoid lines established by Narayan expressed 509 genes that matched genes for a “fibrolamellar signature”. The tumors in NSG mice transplanted with the FLC organoid lines showed FLC characteristics. Thus, the FLC organoid models established by Narayan have the characteristics of patient derived FLC tumor tissues.

However, the Clever`s system normally embedded tumor cells into the Matrigel, this may lead to missing critical features of FLC during the organoids formation. Therefore, different groups had revisited the Patient-Derived Xenograft (PDX) models or the combination of PDX models with organoids models for better presents the features of tumors. 

## 5 PDX models developed for FLC

PDX models, particularly those using serial subcutaneous transplantation in immunocompromised hosts, have proven suitable for modeling FLC, but are unique among transplantable tumors in requiring long passage times on the order of months (4), which means that this experimental approach is time consuming, labor- intensive and expensive (42, 43). Compared to the exclusively animal-based PDX model, the establishment of PDX-derived organoid models is a more cost-effective and tractable approach in the study of human solid tumors.

The first-ever patient-derived PDX model of FLCs, FLC-TD-2010 (4), was developed by Oikawa and Wauthier in the Reid Lab (UNC School of Medicine, Chapel Hill, NC). It was isolated from FLC ascites tumor cells cultured briefly (one or two weeks) in serum-free Kubota’s Medium (KM), designed for endodermal stem/progenitors, and used to select organoids of FLCs that partnered with their associated mesenchymal cell precursors comprised of precursors for endothelia and stellate cells; the organoids were transplanted into immunocompromised hosts (**Figure 3**, **Table 2**) (44). All transplantable FLC tumor lines established with those organoids expressed the FLC-specific fusion gene DNAJB1-PRKACA and were tumorigenic in immune-compromised hosts such as NSG mice. The FLC-TD-2010 model was validated as the first bona fide model of human FLCs and was used subsequently in research of FLCs with respect to their genetic signatures, pathogenesis and treatment strategies (30, 45, 46).

**Table 2:** Summary of gene mutation and genetic signature of FLC.

Genetic signature	Type of mutation	Occurrence in FLC patient	Correlated treatment	Reference
DNAJB1::PRKACA	A heterozygous 400 kb deletion mutation	In almost all FLC patients	DNAJB1-PRKACA fusion kinase peptide vaccine	Joshua N. Honeyman et al., *Science* 2014 Rondell P. Graham et al., *Modern Pathology* 2015 Lars H. Engelholm et al., *Gastroenterology* 2017
PRKAR1A	PRKAR1A inactivating mutation	In FLC patients with a history of Carney complex	PKA inhibitors or immunomodulators	Rondell P. Graham et al., *Hepatology* 2017
BAP1	BAP1 inactivating mutation	More frequently in FLC female patients without chronic liver disease or cirrhosis	PKA inhibitors or immunomodulators	Théo Z Hirsch et al., *Journal of Hepatology* 2020

Later, Lalazar et al. established six FLC-PDX models using tumor tissue from six untreated or chemotherapy-only FLC patients (47). The model verification results confirmed that the DNAJB1-PRKACA fusion gene and its fusion protein can stably express in xenografts after multiple passages. Histological analyses showed that these PDX models had the typical morphological features of FLC, such as eosinophilic cytoplasm and areas of fibrolamellar bands. Additionally, these FLC-PDX models was proven to be outstanding for *in vitro* drugs screening and *in vivo* drug testing. With these PDX models and the organoids generated from these models Lalazar et al. were able to test drugs including napabucasin (a novel STAT3 inhibitor), TOPO1 and HDAC inhibitors on the primary and metastatic FLCs. Their results showed that these drugs have synergistic inhibitory effects with the anti-apoptotic protein Bcl-xL. Based on their study, Lalazar suggested that eliminating oncogenes, oncotranscripts, or oncoproteins can be an effective treatment for FLC.

## 6 Animal models for FLCs

A reasonable and reliable animal model can simulate the microenvironment of human tumors and reflect their cellular and molecular pathological characteristics. It provides a platform to elucidate etiology and screen therapeutic drugs for effective treatments. Currently, animal models, including genetically engineered/modified mouse models (GEM) and larger animal models (especially dogs, pigs, monkeys), are used in scientific research of most tumors, such as liver cancer, pancreatic cancer, and breast cancer (48–50). Not only are GEM models efficient to operate and cost-effective, but they can also exhibit genetic heterogeneity (51). Tumors can occur naturally in a specific microenvironment, which can better simulate the molecular and pathological characteristics of human diseases. It provides a more ideal spectrum for studying the pathological properties of a particular gene *in vivo*.

### 6.1 Animal models of FLCs (zebrafish)

The morphological characteristics of zebrafish larvae are small and translucent, which is an ideal form for imaging (52). Therefore, zebrafish larvae are one of the valuable models for studying the cellular morphology and molecular characteristics of early-stage liver cancer (53, 54). Oliveira et al. overexpressed a pair of homologous fusion genes DNAJB1a::PRKACAa in zebrafish using the hepatocyte promoter *fabp10a* and established a stable Zebrafish line, *Tg(fabp10a:dnajb1a-pekacaa_cryaa:Cerullean) (* 55). By comparing liver morphology with that in normal zebrafish, Oliveira demonstrated that zebrafish with DNAJB1a::PRKACAa overexpression displayed early malignant features, including hepatomegaly, infiltration of immune cells such as neutrophils and macrophages, and activation of caspase-a. Meanwhile, pharmacological inhibition of TNFα secretion and caspase-a with pentoxifylline and Sc-YVAD-CMK, respectively, was investigated in the liver of FLC-zebrafish, and both were found to reduce immune cell inflammation and hepatomegaly in the FLC progression. Therefore, this study suggests that TNFα and caspase-a may represent novel targets for limiting FLC progression.

### 6.2 Animal models of FLCs (mouse)

Mouse models are important tools for assessing the carcinogenic potential of candidate cytokines and exploring the mechanism of tumorigenesis (56). Kastenhuber et al. also used CRISPR-mediated endogenous gene deletion to create a C57 mouse model with DNAJB1-PRKACA fusion gene mutation. CRISPR.1 and CRISPR.2 guide RNA were used for gene editing in the liver of adult mouse, respectively. It was the first time that the FLC model of mature mouse liver expressing the DNAJB1-PRKACA fusion gene was constructed. Liver tumors leading to a moribund condition were observed in the gene-edited mice 16 to 24 months after injection. The liver tumors of these model mice had the characteristics of human FLC tumor tissue, but did not express the cholangiocytic markers CK7, CK19 and CD68. It is hypothesized that this may be because the way liver tumorigenesis is achieved in mice using gene editing is different from that in humans. In addition, tumor molecular profile analyzes showed that proliferation and mitogenic signaling pathways were enhanced in FLC tumor cells, and the activation of the WNT signaling pathway cooperated with the expression of DNAJB1-PRKACA to accelerate FLC formation. Furthermore, the tumorigenicity of the DNAJB1-PRKACA fusion gene was found to be mainly dependent on the kinase domain of PRKACA.

Furthermore, Engelholm et al. designed a PX330 recombination vector that coexpresses Cas9 protein and gRNA. It was then injected into female FVB/N mice at approximately 8 weeks via hydrodynamic tail vein injection (17). In the experimental group of mice without mutagenic agents, the proportion of liver tumor formation was about 80%, and the mice with FLC tumors showed features similar to human FLC tumors, such as the increase of cell size and intracellular mitochondria. Therefore, it was suggested that expression of the fusion gene could induce the formation of FLC tumors in mice.

Although sufficient studies have shown that the DNAJB1-PRKACA fusion gene can induce FLC formation in mice, the downstream signal transduction process is still unclear. Transcriptome analyses revealed that the non-coding RNA expression profile in FLC tumor tissues is significantly different from that of adjacent normal liver and other liver tumors. The studies have provided ideas for exploring other possible tumorigenic factors in FLCs. Farber et al. identified the miRNA and lncRNA expression in FLC. The lncRNA expression profile is distinctly different from the normal liver and other liver tumor tissues. This proved that these changes in the cellular levels of miRNA are correlative with tumorigenesis of FLC (57). Similarly, Sethupathy and his associates found that expression of the DNAJB1-PRKACA fusion gene inhibits the expression of miRNA-375 and then targets YAP1 and connective tissue growth factor (CTGF) in the Hippo signaling pathway, leading to increased proliferation and invasion of FLC cells (30). Therefore, their results suggest that miRNA-375 may suppress the growth of FLC (30). The therapeutic strategy based on this is promising.

In summary, the mouse model established by Engelholm and Kastenhuber is easy to implement and reproducible and does not require the costly and time-consuming process of generating and breeding mouse strains. Therefore, it could be an effective model for further studying of the biological properties of FLC. However, as Weinberg said, “mice are not small people”, and these models can not accurately simulate all the characteristics of human diseases (58). Knocking out a gene in an organism by using gene editing can have complex consequences. Due to several confounding factors, it is impossible to precisely understand the specific function of the fusion gene that is central to FLC pathology. Before selecting the most ideal model for drug screening or new treatment innovations, a balance between feasibility and prevention of tumor function in different models should be assessed overall.

## 7 New treatments

Currently, surgical resection is the primary treatment for early-stage FLC patients, with surgically treated individuals having a higher survival rate (6). However, because FLC is a primary cancer without typical signs of liver damage, early predictive signals and clinical symptoms are lacking (59). There is still no standardized, effective, and systematic treatment(s) for patients with advanced FLC disease. Chemotherapeutic drugs such as gemcitabine, oxaliplatin (GEMOX), which are cisplatin and sorafenib, used in the treatment of hepatocellular carcinoma have also been used in targeted chemical therapy in patients with advanced FLC disease, but tumors had limited response to these drugs. The proliferation and metastasis of FLC tumors could not be inhibited (6). Currently, patients diagnosed with FLC are enrolled in the Pediatric Hepatic Malignancy International Therapeutic Trial (PHITT) to receive surgery in combination with cisplatin and doxorubicin (**Table 3**). If patients are not suitable for surgical resection, they are treated periodically with sorafenib, gemcitabine, and oxaliplatin (NCT03533582). The use of immunotherapy remains a future approach for effective treatments for FLC, and studies are underway to define logical immunotherapeutic protocols. The combination of precise immunotherapy directly targeting the FLC oncoprotein and comprehensive immune checkpoint blocking can alter the key regulatory pathways of FLCs and help improve the systemic therapeutic effect of FLCs.

**Table 3:** Current clinical trials of FLCs and the latest updates.

NCT#	Description of treatment	Current outcomes	Limitations
NCT02234986	Oral ENMD‐2076 for the Treatment of Patients with Advanced Fibrolamellar Carcinoma.	Of 35 patients who enrolled and received treatment, 1 (3%) had a partial response and 20 (57%) had stable disease. Three deaths were reported on-study—two due to disease progression and one due to pulmonary embolism not related to ENMD-2076.	At present, there is no comprehensive therapeutic approach supporting the further evaluation of ENMD-2076 as a single agent.
NCT01642186	A Randomized Three Arm Phase II Study of (1) Everolimus, (2) Estrogen Deprivation Therapy (EDT) With Leuprolide + Letrozole and (3) Everolimus + EDT in Patients with Unresectable Fibrolamellar Hepatocellular Carcinoma	Stable disease was observed in 9 of 26 evaluable patients (35%). PFS6 was 0%. Median overall survival (OS) was 12.4 months.	There are side effects such as nausea, vomiting, anemia, and elevated aspartate aminotransferase.
NCT05014607	DNAJB1-PRKACA neoepitope-based personalized peptide vaccine adjuvanted with the TLR1/2 agonist XS1532 and MontanideTM ISA51 VG in a single FL-HCC patient.	Patients can survive recurrence-free survival for more than 21 months after vaccination.	No released information
NCT04248569	DNAJB1-PRKACA Fusion Kinase Peptide Vaccine Combined with Nivolumab and Ipilimumab for Patients with FLC	Pending	No released information
NCT04380545	To evaluate the safety and tolerability of therapy with nivolumab + fluorouracil (5-FU) + recombinant interferon alpha 2b-like protein (IFN-alpha2b) in patients with unresectable FLC in the context of palliative systemic and prebiopsy therapy.	Pending	No released information

The exploration of new therapeutic targets and the realization of an effective treatment is one of the current topics of FLC research. Early clinical treatments of FLC mainly focus on chemical drugs of renal cell carcinoma, hepatocellular carcinoma, and other tumors, such as Sunitinib, ENMD-2076 and other oral drug treatments. However, the therapeutic effects found so far are not ideal. According to the recent studies on the phenotypic characteristics and specific markers of FLC (13, 16), clinical trials of combined drug therapy and cellular immunotherapy for FLCs have been conducted in the past two years. For example, Nivolumab, Fluorouracil and interferon-α-2B (NCT04380545), the Glutamine Antagonist DRP-104 combined with Durvalumab (NCT06027086), and the DNAJB1-PRKACA fusion kinase peptide vaccine in combination with Nivolumab and Ipilimumab (NCT04248569) have been used in the treatment of advanced FLC patients. These clinical trials are still in the volunteer recruitment phase.

## 8 Glycosaminoglycan biology and FLCs

New areas of research for FLCs include glycosaminoglycan (GAG) chemistry and its regulation of FLC organoids through complexes of paracrine signals and specific GAG oligosaccharides. The ability to perform such studies is due to revolutionary breakthroughs by a team of chemists, Jian Liu (School of Pharmacy, UNC, Chapel Hill, NC). Liu have established strategies for the synthesis of chondroitin sulfate-(CS)-oligosaccharides and heparan sulfate-(HS)-oligosaccharides. It has long been known that complexes of GAG oligosaccharides and specific proteins have dramatic regulatory effects on cell growth and differentiation. However, in the past the effects of GAGs could not be studied given the hundreds of variant chemistries extant among CS- and HS-oligosaccharides present in extracts. With synthesis of large quantities of each unique CS- or HS-oligosaccharide, one can do research on their cellular and molecular effects when in a complex with a specific protein. Jian Liu and his associates have collaborated with the molecular geneticists in Praveen Sethupathy’s lab and, in parallel, with the cell and molecular biologists in the Reid lab to compare the GAG oligosaccharides in FLCs versus normal tissues and then analyzed the biological effects of some of the synthesized oligosaccharides on FLCs.

CS-oligosaccharides are sulfated GAGs comprised of disaccharides of glucuronic acid (GlcA) or iduronic acid (IdoA) and sulfated galactosamine and its associated proteoglycans, such as versican (VCAN). They were examined in normal livers compared to FLCs to determine their relative quantities. It was found that CS-oligosaccharides (but not HS-oligosaccharides) are dramatically more abundant (6-fold), and the expression index of VCAN, secreted by activated stellate cells, is significantly higher in FLC tumors as compared to normal livers. The implications are that CS-oligosaccharides and their associated proteoglycans, especially those from activated stellate cells, are a striking feature of FLCs (60). Future research will focus on assessment of the effects of complexes of specific CS-oligosaccharides and paracrine signals on organoids of stem cell subpopulations compared to FLCs compared to adult hepatocytes.

HS-oligosaccharides are sulfated glycosaminoglycans (GAGs) from the disaccharides of glucuronic acid (GlcA) or iduronic acid (IdoA) and sulfated glucosamine. HS-oligosaccharides bind to core proteins to form HS-proteoglycans (HS-PGs). Hormones or paracrine signals bind tightly to the HS-oligosaccharides on those HS-PGs and together they form three-dimensional structures that bind to receptors triggering signal transduction resulting in various cellular functions (61–64). The biological effects of HS-oligosaccharides depends on their complex sulfation motifs that dictate their binding to specific signaling proteins and that in turn to the presentation of the complex to cell receptors that trigger signal transduction (65, 66).

The effects of synthesized HS-oligosaccharides and paracrine signaling complexes on FLC organoids were examined and compared with normal BTSC organoids or HpSCs organoids (66, 67). The organoids divided steadily with a division every approximately 7 days. When spheroids were prepared, from organoids by eliminating the mesenchymal cells within, the cells the spheroids survived indefinitely in a condition of growth stagnation for several months. The mesenchymal cells, precursors to endothelia and to stellate cells, were shown to be the source of multiple paracrine signals such as fibroblast growth factors (FGFs), epidermal growth factors (EGFs), vascular endothelial growth factors (VEGFs), and Wnt ligands, etc. Distinct HS-oligosaccharides, all of them 10-12 mers or larger, could form complexes with the various paracrine factors, and each complex was able to elicit particular biological responses that proved distinct between the FLC organoids versus organoids of BTSCs or HpSCs. Some of the complexes, especially those with 3-*O* sulfated HS-oligosaccharides were able to cause the FLC organoids to go into growth arrest for weeks.

In the analyzes of the more than 50 distinct HS-oligosaccharides synthesized by Jian Liu and his associates, the HS-oligosaccharides with biological activity on the organoids were all 10-12 mers and larger. They were tightly bound to the various paracrine signals, and the complexes were found to be biologically effective on organoids of all stem cell subpopulations (66). Among the most potent proved to be those HS-oligosaccharides with 3-*O*-sulfation, a rare modification. However, this finding of 3-*O*-sulfated HS-oligosaccharides potent effects on organoids of normal and transformed hepato/pancreatic stem cells, parallels their potent biological effects in the treatment of coagulation disorders (68) and for the expansion of normal stem cells (69). In contrast to classical signal transduction pathways that are triggered only by proteins, those that are regulated by complexes of proteins and HS-oligosaccharides cannot be replaced by other alternative pathways. Furthermore, synthesized HS-oligosaccharides can be synthesized into compounds that are insensitive to heparanase, which may provide a novel and effective treatment for FLC in the future.

## 9 Conclusions and prospects

Although the newly discovered therapeutic targets provide new ideas for the treatment of FLCs, significant work is still needed to elucidate them. In addition, attention must be paid to the efficiency of medication administration. FLC tumors are enveloped in thick fibrolamellar bands that contain an abundance of extracellular matrix that can protect the tumor cells from various therapeutic modalities. The FLCs may exhibit features of stem cells from either the hepatic or pancreatic (or both) lineages, meaning that there will be variability in key features of FLCs depending on whether oncogenic transformation occurred in lineage stages within the biliary tree nearer to the liver versus pancreas. These variabilities are ones yet to be adjudicated in some of the ongoing research. For example, those located in the branches that can differentiate into cells with pancreatic characteristics can produce large amounts of pancreatic exocrine enzymes and matrix metalloproteinases, which pose major challenges for drug delivery and stability.

Nevertheless, there have been gratifying advances in the study of FLCs in terms of genetic and protein signature studies as well as analyzes in several *ex vivo* and *in vivo* models. There are still no fully validated treatments for FLC patients beyond surgical removal of tumors in patients with a non-metastatic tumor. Fortunately, there are multiple research directions with promising insights into novel treatments for the future, particularly in some of the ongoing research that are analyzing forms of immunotherapies.
